# Establishment of a Jaw Fibrosarcoma Patient-Derived Xenograft and Evaluation of the Tumor Suppression Efficacy of Plumbagin Against Jaw Fibrosarcoma

**DOI:** 10.3389/fonc.2020.01479

**Published:** 2020-08-27

**Authors:** Yuqi Xin, Shiya Li, Qingkun Jiang, Fangling Hu, Yuanqiao He, Jie Zhang

**Affiliations:** ^1^Department of Oral and Maxillofacial Surgery, The First Affiliated Hospital of Nanchang University, Nanchang, China; ^2^Medical College, Nanchang University, Nanchang, China; ^3^Department of Otolaryngology Head and Neck Surgery, The First Affiliated Hospital of Nanchang University, Nanchang, China; ^4^Laboratory Animal Science Center of Nanchang University, Nanchang, China; ^5^Key Laboratory of Experimental Animals of Jiangxi, Nanchang, China; ^6^Nanchang Royo Biotechnology, Nanchang, China

**Keywords:** jaw fibrosarcoma, patient-derived xenograft (PDX), histopathological assessment, plumbagin, Ki67

## Abstract

**Background:** Head and neck fibrosarcoma is a rare malignant tumor, accounting for about 1% of all head and neck tumors. It can also occur in the jaw bone, for which surgical resection is the main treatment but the recurrence rate is high and the prognosis is usually poor. Due to the lack of models mimicking the biological characteristics of the tumor, there is little progress in the research of the pathogenesis and treatment of fibrosarcoma. Therefore, there is an urgent need to explore a high-fidelity model that can reflect the biological characteristics of fibrosarcoma for the sake of improving the therapeutic outcome and prognosis, and preventing recurrence. Patient-derived xenografts (PDX) may more accurately reflect the human disease, and is an attractive platform to study disease biology and develop treatments and biomarkers. In this study we describe the establishment of jaw fibrosarcoma PDX models and compare PDX tumors to those of human origin.

**Methods:** Tumor biopsies from a patient with jaw fibrosarcoma were implanted in immunodeficient mice. Primary and PDX tumors were characterized extensively by histology, immunohistochemistry and humanized identification. Based on the finding of our previous preliminary research that plumbagin had an anti-tumor effect against head and neck cancer, we used this model in the present study to evaluate the anti-tumor effect of plumbagin on jaw fibrosarcoma.

**Results:** The established PDX model maintained the histological and immunohistochemical characteristics of the primary tumor. Plumbagin significantly inhibited the tumor growth in the jaw fibrosarcoma PDX model.

**Conclusion:** We successfully established a PDX model of jaw fibrosarcoma and demonstrated that this PDX model preserved the important molecular characteristics of the human primary tumor, thus providing a powerful tool for treatment research and new drug development of jaw fibrosarcoma. In addition, plumbagin was found to have an inhibitory effect on the growth of PDX modeled jaw fibrosarcoma, which provides a preliminary research basis for its clinical application.

## Background

Fibrosarcoma is a rare fibroblastic malignancy, accounting for ~5–6% of all adult soft tissue sarcomas (1–4) and about 1% of all head and neck malignancies (5), with the highest incidence occurring between 20 and 40 years of age (6). It is reported that fibrosarcoma can also occur in any part of the jaw bone, and is rarer in the jaw. According to the site of occurrence, jaw fibrosarcoma is classified as the peripheral and central types (7, 8). Surgical resection is the mainstay of treatment for jaw fibrosarcoma, with an overall survival (OS) rate of 45–82% (9–12) due to the high rate of recurrence (13). The prognosis has not improved significantly over the past 20 years (14). Due to the lack of models mimicking the biological characteristics of the tumor, there is little progress in the research of the pathogenesis and treatment of jaw fibrosarcoma. Therefore, there is an urgent need to explore a high-fidelity model that can reflect the biological characteristics of jaw fibrosarcoma for the sake of improving the therapeutic outcome and prognosis, and preventing recurrence.

The patient-derived xenograft (PDX) model is a tool of functional diagnosis recommended in the National Cancer Institute (NCI) guidelines (15). The PDX model is a transplanted tumor model formed by implanting tissue blocks, circulating tumor cells and primary cells derived from tumor patients into immunodeficient mice (16). The PDX model can grow in the environment provided by the animal and at the same time preserve the histological and genetic characteristics of human primary tumors, maintain the heterogeneity and microenvironment of human tumors, and retain their biological characteristics of growth and metastasis (17), thus providing a good pre-clinical evaluation platform for tumor research. In addition, the drug sensitivity test using the PDX model has a clinical correlation of about 90% (18), indicating that the PDX model is a powerful tool for individualized precise cancer therapy and development of new drugs.

Plumbagin is an anthraquinone component isolated from the rhizome of Plumbago zeylanica L. and has anti-tumor effects (19). Our and other research teams have conducted a series of preliminary research on plumbagin (20–24), and demonstrated that plumbagin has an anti-tumor effect against head and neck cancer. We first successfully established a PDX model of human jaw fibrosarcoma and administered drugs in this established model to evaluate the anti-tumor effect of plumbagin against human jaw fibrosarcoma, hoping that the results could provide an experimental foundation for the clinical application of plumbagin.

## Materials and Methods

### Patient and Tissue Samples

**Figure 1:**
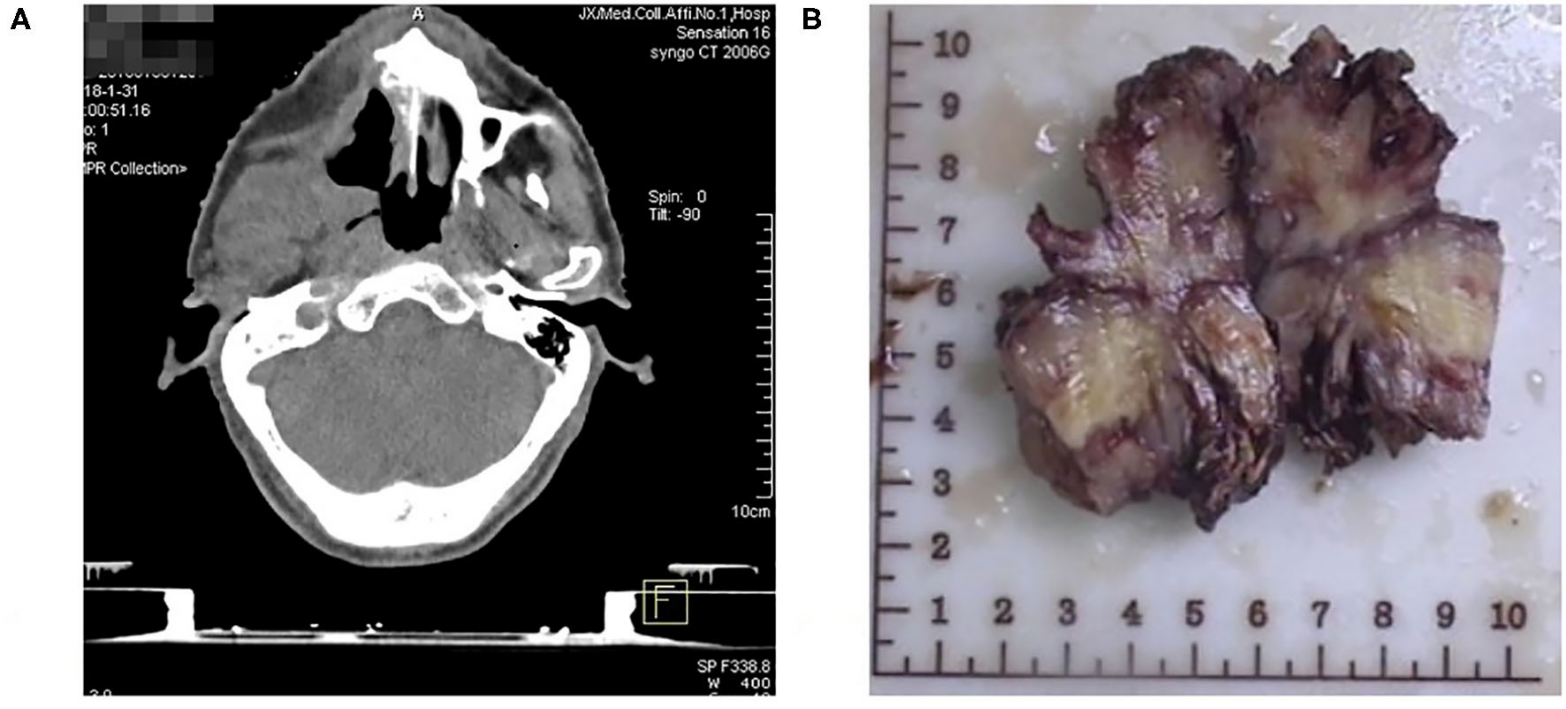
**(A)** CT showing right jaw bone destruction and formation of a soft tissue tumor invading the right pterygoid process and pterygoideus medialis and lateralis muscles. **(B)** Photograph showing Patient's jaw fibrosarcoma specimen.

A 58-year-old man who began feeling pain in the right upper jaw bone 3 years ago, when CT scan showed right jaw bone destruction and formation of a soft tissue tumor invading the right pterygoid process and pterygoideus medialis and lateralis muscles, which was suspected as a maxillary malignant tumor. It was surgically removed after 3 months (Figure 1). Postoperative pathology confirmed the diagnosis of fibrosarcoma.

### Materials

Balb/c nude mice aged 6–8 weeks (Nanjing University-Nanjing Institute of Biomedicine, Nanjing, Jiangsu, China; Certificate No. SCXK 2015-0001) were used for PDX establishment and treatment research. They were reared in an SPF environment. All animal-related procedures were performed according to the protocols approved by Institutional Animal Care and Use Committees of Nanchang University (Permit No. SYE2019101905).

Plumbagin (Lot# SLBS8515) was purchased from Sigma-Aldrich (China). Cisplatin (H20040813) was purchased from Haosen Pharmaceutical Co., Ltd. (Jiangsu, China).

### Establishment of a PDX Jaw Fibrosarcoma Model

The fresh surgically resected jaw fibrosarcoma tissue was cut into 2 mm × 2 mm × 2 mm small pieces and inoculated subcutaneously at the mouse scapula aseptically in <24 h. This inoculated mouse was numbered as P0. When the tumor volume grew to more than 1,000 mm^3^, the tumor was passaged to P5 by the same method and used for subsequent experimental research.

The tumor size was measured using a vernier caliper when the inoculated tissue grew into the tumor. The tumor volume (mm^3^) was calculated by the following formula: V = a × b^2^/2, where V represents the tumor volume, and a and b are the longest and shortest tumor diameters, respectively.

### Histopathological Assessment

The patient's tumor tissue and PDX were fixed in formalin, paraffin-embedded, prepared into 4 μm dry slides, and stained with hematoxylin-eosin (H&E) using an H&E staining kit (Applygen, Beijing, China) according to the manufacturer's instructions. Immunohistochemical (IHC) staining was performed using specific antibodies, including VIM (Abcam # ab92547), CD68 (Abcam # ab955), CD34 (Abcam # ab8158), S100β (Proteintech # 15146-1-AP), and Ki-67 (Abcam # ab15580).

### Polymerase Chain Reaction (PCR)

Nucleic acid extraction was performed at low temperature in PDX model tumor tissues. Primer5.0 primer design software was used to design human- and mouse-derived genome-specific primers (Table 1). The extracted sample was amplified by PCR. Five microliter DNA Marker (DL2000) was added as a reference for the length of the gene amplification fragment. 1 × TAE buffer was added to the electrophoresis tank for reference. Electrophoresis was performed at 100 V voltage for 15 min. When the indicator bromophenol moved to 2/3 of the gel, electrophoresis was terminated. The amplified fragment was observe in the DNA gel electrophoresis imager and photographed for analysis.

**Table 1:** DNA primers.

**Primers**	**Sequences**	**PCR products (bp)**	
mus-F1	CAGGTTGTCTCCTGCGACTT	571 bp	Mouse genome specific primer 1
mus-R1	CAGCTGGATGTCAGAGCCAA		
mus-F2	AAGGGCATCTTGGGCTACAC	549 bp	Mouse genome specific primer 2
mus-R2	CCTGCTTCACCTCCCCATAC		
hs-F1	GGCTCTTAAAAAGTGCAGGGTC	327 bp	Human genome specific primer 1
Hs-R1	ATGGTACATGACAAGGTGCGG		
hs-F2	TAACTGTCTGCTTCTCTGCTGTAGGC	772 bp	Human genome specific primer 2
Hs-R2	GCTTCACCACCTTCTTGATGTCATCA		

### Treatment

The P5 generation PDX model was used for the experiment. Drug administration was initiated when the tumor volume reached 100–200 mm^3^. Thirty-two mice were equally randomized into four groups: (A) blank control group; (B) cisplatin (CDDP) group, 5 mg/kg, intraperitoneal (i.p.) injection once a week; (C) plumbagin (PLB) group, 2 mg/kg, per i.p. injection (24); (D) plumbagin + cisplatin group. The drug dosage and administration were the same as before. Body weight (BW) and the subcutaneous tumor size were measured twice a week. After 21-day drug administration and a week after drug discontinuation, the animals were sacrificed. The tumors were excised and weighed. Each tumor tissue was cut into two halves. One half was fixed in formaldehyde and the other half was stored at −80°C. And major organs including the heart, liver, spleen, lung, and kidney were removed and fixed in formaldehyde for histopathological assessment.

### Western Blotting

First of all, the tissue protein of the PDX jaw fibrosarcoma model was extracted, and the protein content was determined using a BCA Protein Assay Kit (CW0014S, Kangwei Century Biotechnology Co., Ltd., Beijing, China). Then, the tissue protein sample was separated by SDS-PAGE and transferred to the polyvinylidene fluoride (PVDF) membrane (IPVH00010, Millipore, Billerica, MA, USA). The membrane was blocked with 3% skim milk (P1622, Pulilai Gene Technology Co., Ltd., Beijing, China) and incubated with the primary antibody overnight. After washing, the membrane was incubated with the secondary antibody for 2 h. Finally, the PVDF membrane was soaked with luminescent liquid (RJ239676, Thermo Fisher Scientific, Waltham, MA, USA) and placed in an ultra-high sensitivity chemiluminescence imaging system (Chemi DocTM XRS+, Bole Life Medical Products Co., Ltd., Shanghai, China) for image development.

### Statistical Analysis

All calculations were performed by SPSS 22.0 (IBM Corp. Armonk, NY). Results are expressed as the means ± standard deviation (SD). Comparison between two groups was conducted by *t*-test and one-way analysis of variance (ANOVA). Comparison between multiple groups was conducted by Tukey's *post-hoc* test. *P* < 0.05 was considered to indicate a statistically significant difference.

### Ethics Approval Statement

All experiments using immunodeficient mice were carried out in accordance with the guidelines approved by the Institutional Animal Care and Use Committees of Nanchang University (Permit No. SYE2019101905). Written informed consent was obtained from all patients and the study was approved by the First Affiliated Hospital of Nanchang University Ethics Committee (Permit No. 2019017).

## Results

### Establishment and Identification of the PDX Jaw Fibrosarcoma Model

**Figure 2:**
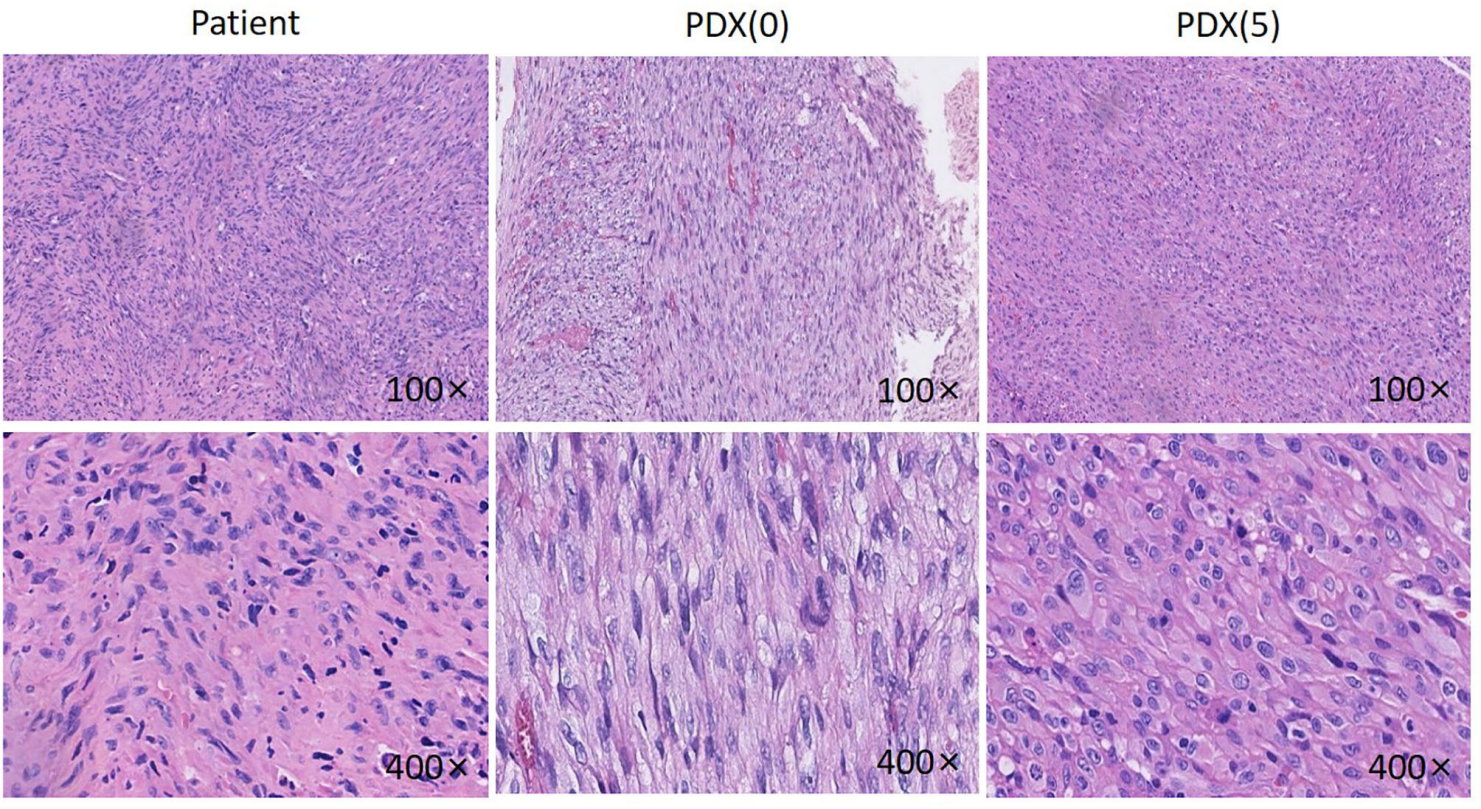
HE pictures of the patient, P0 generation PDX and P5 generation PDX. Histological analysis of tumor samples. After sacrificing the mice, Jaw fibrosarcoma tissues from patient, P0 generation PDX and P5 generation PDX were fixed and checked with hematoxylin/eosin-staining. Cell nuclei were stained with hematoxylin (purple).

The PDX model was established in nude mice and appeared ~3 months after implantation, whereby passage was carried on successfully for other analyses. Comparison of H&E and IHC staining between the patient tumor and the subsequent passages was performed by a pathologist accredited by a committee specializing in head and neck cancer (RH). Evaluation of the patient's tumor showed that the tumor consisted of fusiform fibroblast-like cells (Figure 2). The cell nuclei were densely stained, the chromatin was coarse, the cytoplasm was sparse, the eosin was red, and the cell boundary was not clear. Well-differentiated spindle cells were arranged in a classic “fish-like” or “herringbone” structure, with small differences in cell size and morphology, but with varying degrees of nuclear division and formation of large numbers of collagen fibers, while poorly differentiated cells were arranged densely with irregular morphology in an obese and round or oval shape with significant cell morphism, more cleavage activity, fewer collagen fibers, and moderate differentiation between the two.

The established P0 and P5 PDX tumors were H&E stained and evaluated (Figure 2). The PDX model tumor was mainly composed of fibroblast-like cells, with deep nuclear staining, coarse chromatin, less cytoplasm, eosinophilia, and unclear cell boundaries, showing a growth pattern and morphology similar to the original tumor.

**Figure 3:**
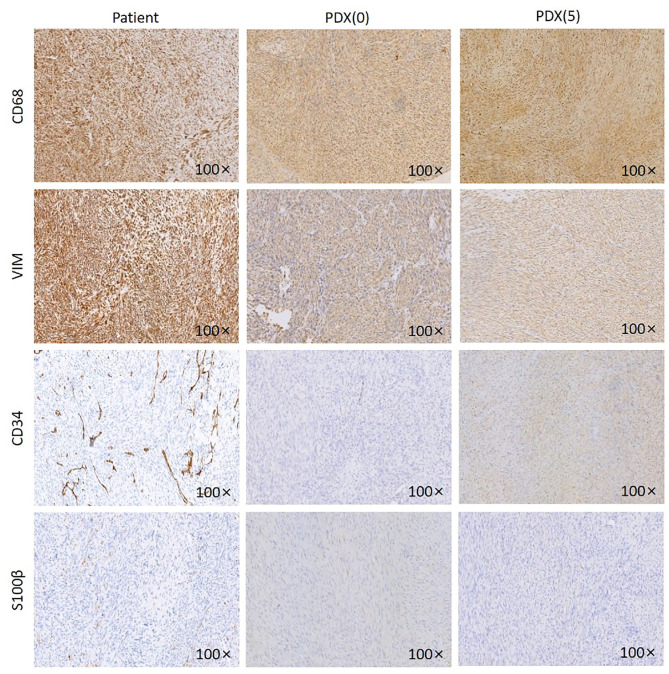
IHC images of the patient, P0 generation PDX and P5 generation PDX. Jaw fibrosarcoma tissues from patient, P0 generation PDX and P5 generation PDX were fixed and performed immunohistochemistry using specific antibodies, including CD68, VIM, CD34, and S100β. Magnification ×100.

Immunohistochemistry of VIM, CD68, CD34, S100β was performed on patient samples and P0 and P5 generation PDX models (Figure 3). The established PDX models maintained the immunohistochemical characteristics of the patient fibrosarcoma. The results were as follows: CD68 (+), Vim (+), CD34 (–), and S100β (–).

Overall, the characteristics of the established PDX model were concordant with those of the primary tumor histologically and immunohistochemically.

### The Established PDX Model Is a Human-Derived Model

**Figure 4:**
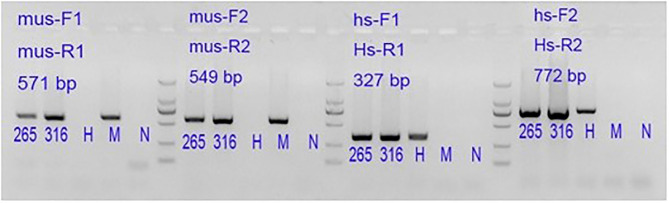
265: P0 generation PDX, 316: P5 generation PDX. Lane 1#: tissue sample; Lane H#: human blood genomic DNA (human genome positive control); Lane M#: B6 mouse genomic DNA (mouse genome positive control); Lane N#: NTC, No-Template Control is a template-free control; DL2000 Marker: 2,000 bp\1,000 bp\750 bp\500 bp\250 bp\100 bp.

The genomic DNA was extracted from the established P0 and P5 tissues by PCR and analyzed by electrophoresis (Figure 4). The tested issue samples contained both mouse and human-derived genes. It shows that the xenotransplantation model we established originates from human tissue.

### The Anti-tumor Effect of Plumbagin on Jaw Fibers

**Figure 5:**
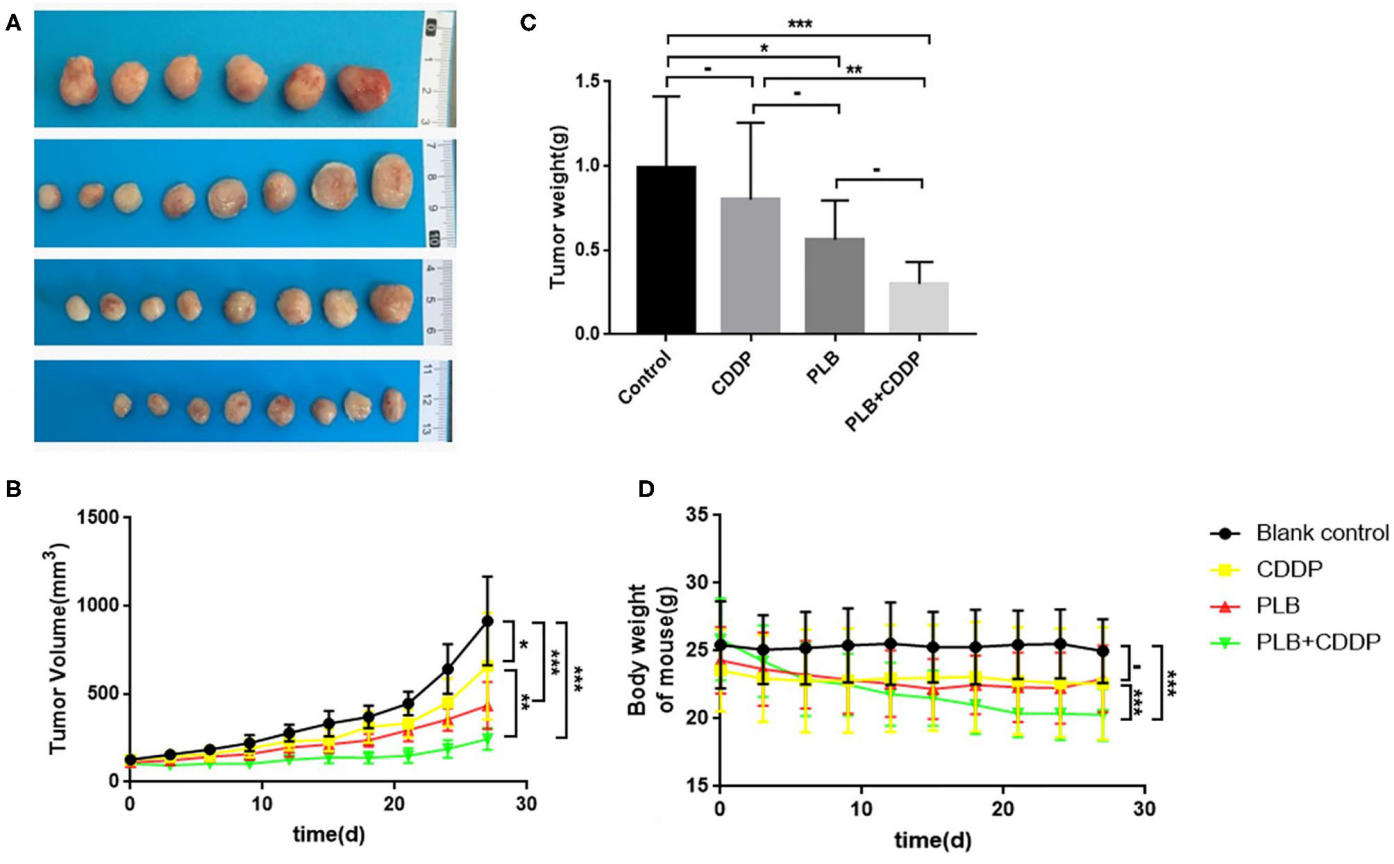
The inhibitory effect of plumbagin on jaw fibrosarcoma tumor growth in PDX model. Patient-derived tumors were subcutaneously established in nude mice. **(A,B)** When the tumors reached 100–200 mm^3^ in size, mice were treated with drugs for 3 weeks and a week after drug discontinuation. And representative PDX samples were resected (eight tumors per group) showing the difference in tumor volumes and weights between blank control, plumbagin (2 mg/kg), cisplatin (5mg/kg), and combined group. **(C,D)** Tumor sizes and weight of mice were measured every 3 days. *p < 0.05, **p < 0.01, and ***p < 0.001.

**Figure 6:**
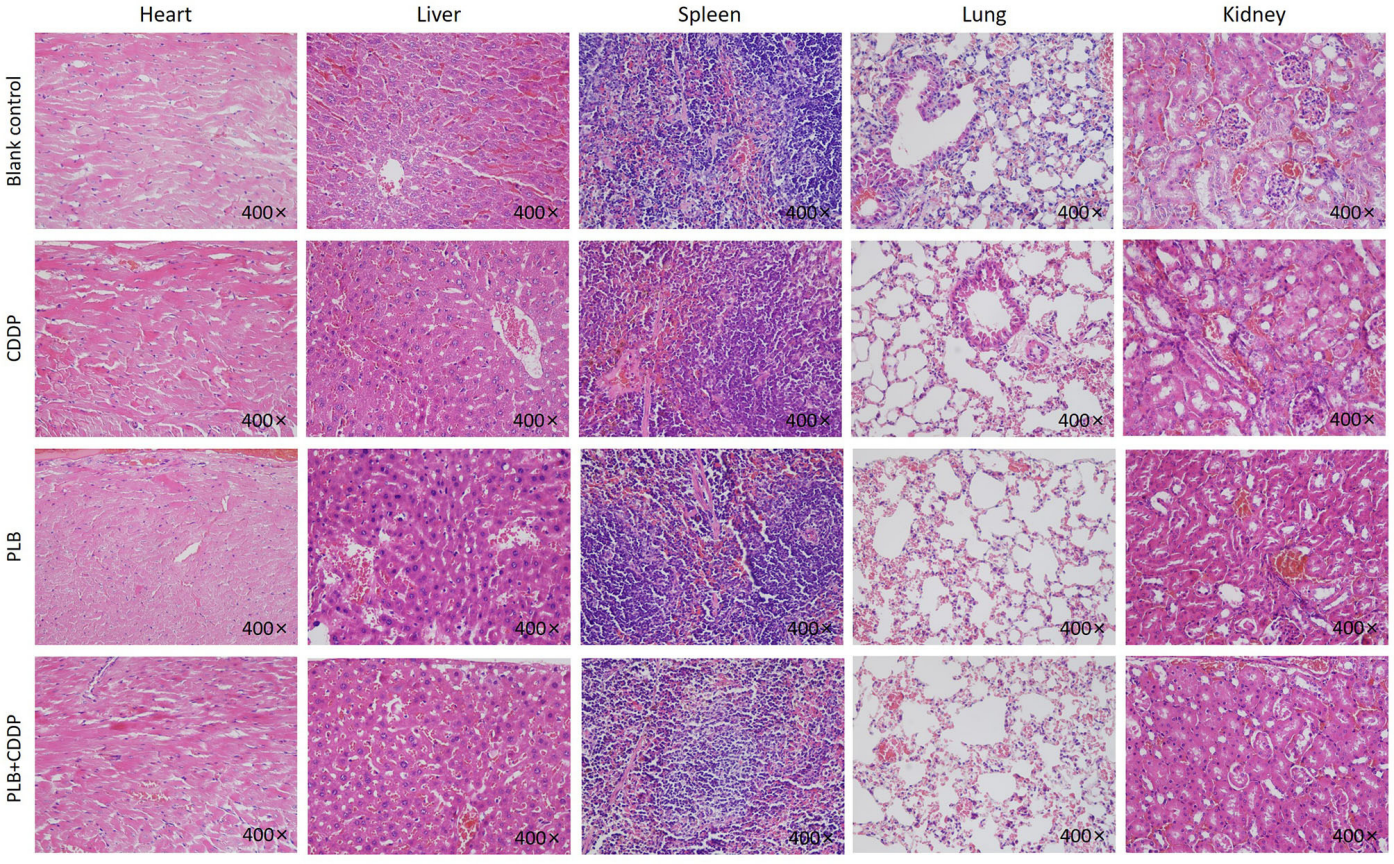
The heart, liver, spleen, lung, and kidney were sectioned and stained with H&E after PLB, CDDP and combined treatment. Magnification ×400.

To investigate the inhibitory effect of plumbagin on tumor growth *in vivo*, patient tumors were implanted to nude mice. The 32 modeled mice were equally randomized to four groups: a blank control group; a cisplatin group [intraperitoneal (i.p.) injection of 5 mg/kg, once a week]; a PLB group (i.p. onjection of 2 mg/kg qd); and PLB+CDDP group, combinational group: both plumbagin and cisplatin were administered according to the aforementioned regimens. After 28 days, an effective inhibitory effect on tumor growth was observed in all treatment groups (Figures 5A–C), especially in the PLB and combined-treatment groups, and the inhibitory effect was the most pronounced in the combined-treatment group. No significant change in body weight was observed in the mice administered with PLB and CDDP compared with the blank control group (Figure 5D), but body weight loss was significant in the combined-treatment group. H&E staining of the heart, liver, spleen, lung, and kidney showed no major organ-related toxicities in all treatment groups (Figure 6).

Overall, we preliminarily judged that the use of plumbagin alone and the combination of plumbagin and cisplatin could inhibit the growth of jaw fibrosarcoma. However, it should be noted that the adverse effects of combined medication may be greater than those of single medication.

### The Anti-proliferation Effect of Plumbagin

**Figure 7:**
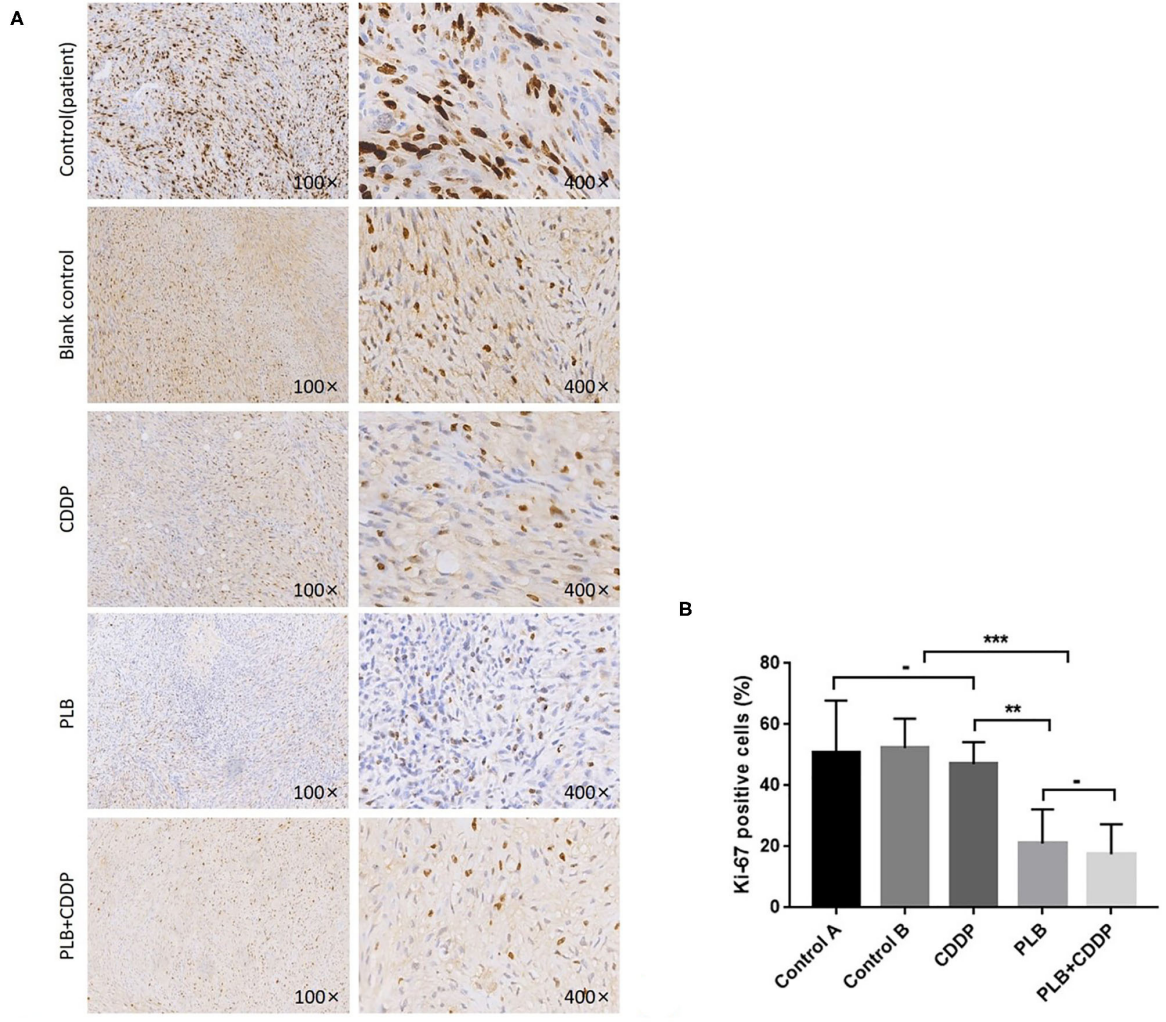
Inhibition of cell proliferation by plumbagin in the PDX model. To check the effect of plumbagin on cell proliferation, immunohistochemical analysis of Ki67 was performed with drug-treated samples. **(A)** Ki67 was stained into brown and nuclei were counterstained with hematoxylin (purple). **(B)** The Ki67-positive cell rate of the patient tissue, blank control group, CDDP group, PLB group, and PLB + CDDP group was 50.8, 52.4, 47, 21, and 17.5%, respectively. ***p < 0.001; **p < 0.01; -, no statistical difference.

**Figure 8:**
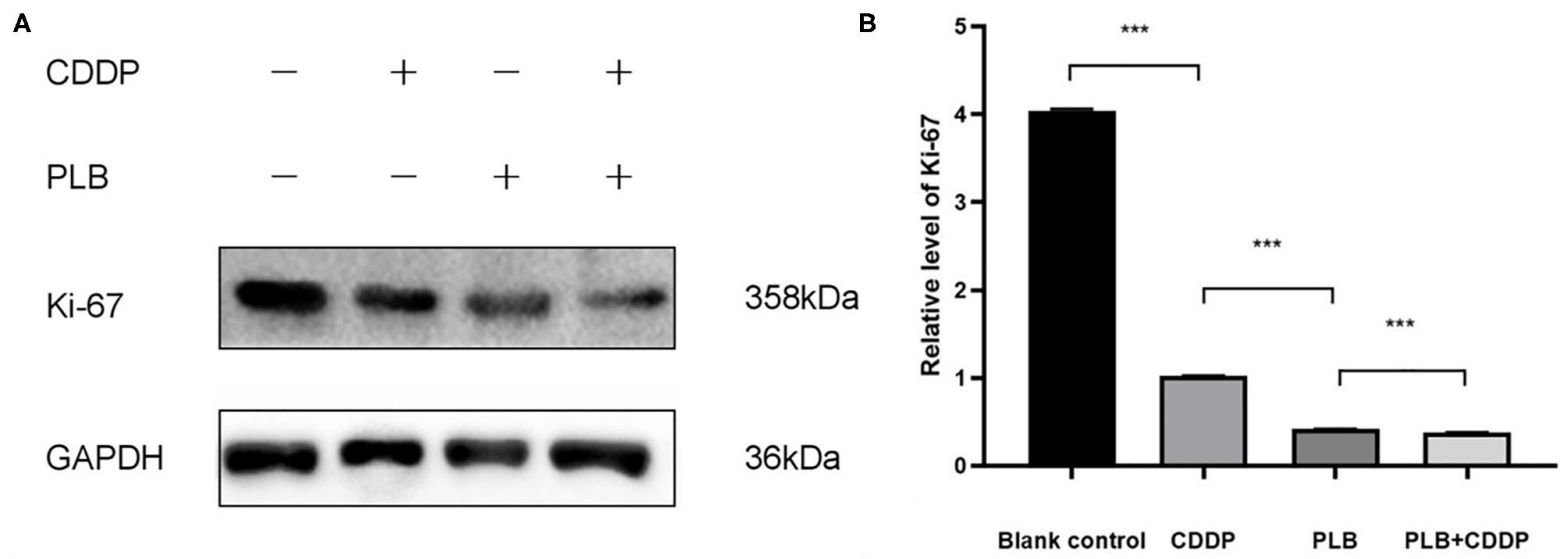
**(A)** The expression level of Ki67 was measured by Western blotting. **(B)** The histograms indicate the relative expression levels of proteins. The quantitative data are shown as the mean ± SD of 3 independent experiments. ***p < 0.001.

To investigate the effect of plumbagin on the proliferation of jaw fibrosarcoma cells, we conducted IHC and Western blotting using the intrinsic proliferation marker Ki67. As shown in Figure 7, the positive rate of Ki67 cells from patient was not statistically different from that in the blank control group, nor was there statistically significant difference in the Ki67 positive cell rate between the cisplatin treatment group and the blank control group, but the detection rate of Ki67 positive cells was significantly higher in the blank control group compared with the tumor tissue of plumbagin and the combination of plumbagin and cisplatin. Western blotting showed that compared with the blank control group and cisplatin group, the expression of Ki67 protein in the plumbagin group and the combined-treatment group was reduced significantly (Figure 8, *P* < 0.05), indicating that plumbagin intervention significantly reduced the expression of Ki67 in the jaw fibrosarcoma PDX model, especially in the combined-treatment group of plumbagin and cisplatin. Taken together, these results suggest that plumbagin inhibited jaw fibrosarcoma growth by decreasing cell proliferation.

## Discussion

Jaw fibrosarcoma is an extremely rare malignancy, the treatment of which remains a formidable clinical challenge due to the lack of preclinical models and effective systemic therapies. In the present study, we established a PDX model of jaw fibrosarcoma, which preserved the histological features after multiple passages, thus making pre-clinical study on jaw fibrosarcoma possible and feasible.

An *in vivo* model is an indispensable procedure for the development of anticancer drugs (25). Compared with traditional cell line-based pre-clinical experiments, the PDX model provides an important tool for tumor research in that it grows in the environment provided by the animal, and at the same time preserves the histological and genetic features of the human primary tumor and maintains the heterogeneity and microenvironment of the patient's tumor, thus better retaining the biological characteristics of the tumor itself including tumor growth and metastasis. The consistency of drug sensitivity between the PDX model and the clinical outcome was more than 90% (18). We have established the jaw fibrosarcoma PDX model is a human-derived model and that has many similarities to the human original. Established PDX models maintain histological and immunohistochemical characteristics. The jaw fibrosarcoma PDX model is a powerful tool for individualized precise cancer therapy and development of new drugs.

Soft tissue sarcoma is a mesenchymal malignancy, and histological biopsy is the gold standard for sarcoma diagnosis. However, inconsistencies often occur between pathologists, so diagnosis is usually done by immunohistochemistry, cytogenetics, and gene sequencing (26). The IHC markers selected in this study were mainly VIM, CD68, CD34, and S100β. VIM vimentin is primarily expressed in mesenchymal cell types, including fibroblasts, bone marrow-derived blood cells, and endothelial cells (27, 28). In this study, we selected VIM because it is a marker of the mesenchymal tissue, suggesting that the tissue is derived from the mesenchyme so that it can be used for the diagnosis of mesenchymal-derived tumors and identification of the PDX model and patient consistency. CD34 molecule is a highly glycosylated type I transmembrane glycoprotein which is selectively expressed on the surface of human and other mammalian hematopoietic stem cells and gradually weakens to disappear as cells become mature. Increased numbers of studies have shown that other than hematopoietic stem cells, many other types of cells also express CD34 molecules, such as certain types of leukemia cells, solid tumor cells, vascular endothelial cells, and fibroblasts (29). CD68 has a wide range of expression and poor specificity, and can be used for the diagnosis of fibrosarcoma (30, 31). S100 protein is closely associated with tumorigenesis (32), knowing that it is highly expressed in malignant fibrous tissue tumors and melanoma (33). Therefore, the IHC markers selected in this study were mainly VIM, CD68, CD34, and S100β. And characteristics of established PDX models were found to be concordant with that of the primary tumor regarding immunohistochemistry.

Plumbagin has a variety of biological properties, including antioxidant, antiviral, antibacterial, antimalarial, and anti-inflammatory activities (24, 34). Some studies have shown that plumbagin has cytoxocity against various cancer cell lines including breast cancer, lung cancer, cervical cancer, leukemia, hepatocellular carcinoma, and oral cancer (34, 35), but its adverse effects were extremely low (19, 24, 36–38). For this reason, it has attracted increased attention and interest from researchers. And our research team have conducted a series of preliminary research on plumbagin (20–22), and demonstrated that plumbagin has an anti-tumor effect against head and neck cancer. In this study, we explored the antitumor effect of plumbagin on jaw fibrosarcoma.

Cisplatin is the first drug widely used to treat solid organ malignancies, including lung cancer, ovarian cancer, testicular cancer, and head and neck cancer (39, 40). However, it has previously been reported to have many serious side effects including nephrotoxicity, neurotoxicity, ototoxicity, nausea, and vomiting (41–45). Many studies have used cisplatin for the treatment of fibrosarcoma (46–48). It was found in our study that plumbagin had better therapeutic effects compared with cisplatin.

Ki67 protein is usually expressed only in proliferating cells (49), and its expression level indicates the state of cell proliferation. Ki67 is highly expressed in most malignant cells but rarely detected in normal cells (50). Increased proliferation is a hallmark of malignant tumors, so Ki67 is considered to be a valuable cancer biomarker. Ki67 has also been proposed as a prognostic marker for cancer (51, 52). In this study, Ki67 was used to preliminarily determine the anti-tumor effect of plumbagin and cisplatin in the PDX model of jaw fibrosarcoma. The results of Ki67 IHC and WB showed plumbagin inhibits jaw fibrosarcoma cell proliferation.

Our previous studies showed that plumbagin could inhibit the growth, invasion and migration of head and neck cancer by inhibiting PI3K/Akt/GLUT1, p38 MAPK, and PI3K/Akt/mTOR-mediated pathways (20–22). In this study, we demonstrated the inhibitory effect of plumbagin on the jaw bone fibrosarcoma, though the specific inhibitory mechanism needs to be further explored.

## Conclusion

In this study, we successfully established a PDX model of jaw fibrosarcoma and proved its consistency with the clinical patients, thus providing a powerful tool for treatment research and new drug development of jaw fibrosarcoma. In addition, plumbagin had an inhibitory effect on the growth of the PDX model of jaw fibrosarcoma, which provides a preliminary research basis for its clinical application.

## Data Availability Statement

All datasets generated for this study are included in the article/Supplementary Material.

## Ethics Statement

The studies involving human participants were reviewed and approved by the First Affiliated Hospital of Nanchang University Ethics Committee (No. 017). The patients/participants provided their written informed consent to participate in this study. The animal study was reviewed and approved by Institutional Animal Care and Use Committees of Nanchang University (RYE2019101905). Written informed consent was obtained from the individual(s) for the publication of any potentially identifiable images or data included in this article.

## Consent For Publication

Informed consent was obtained from patient and his relatives.

## Author Contributions

All authors contributed in the establishment of Model. QJ and SL were responsible for data analysis. YX and FH wrote the manuscript. JZ and YH supervised the work.

## Conflict of Interest

The authors declare that the research was conducted in the absence of any commercial or financial relationships that could be construed as a potential conflict of interest.

## Abbreviations

PDX: Patient-derived xenografts
OS: overall survival
NCI: National Cancer Institute
CT: computerized tomography
H&E: hematoxylin-eosin
IHC: Immunohistochemical
BW: Body weight
RH: head and neck cancer
WB: Western blotting
PLB: Plumbagin
CDDP: Cisplatin.
